# The Hospital Movement in the United States

**Published:** 1905-10-14

**Authors:** 


### HOSPITAL ADMINISTRATION.

### CONSTRUCTION AND ECONOMICS.

### THE HOSPITAL MOVEMENT IN THE UNITED STATES.

### THE ASSOCIATION OF HOSPITAL SUPERINTENDENTS.

The Seventh Conference of this Association was held at Boston, Massachusetts, September 26 to 29 inclusive, under the presidency of Dr. George H. M. Rowe, President of the Association, and medical superintendent of the Boston City Hospital. The membership of the Association may be active or honorary, and "the former must at the time of election be the executive head of a hospital, without reference to sex or denomination." The object of the Association is "the meeting together at stated times of those in immediate charge of hospitals, for the interchange of ideas, comparing and contrasting methods of management, the discussion of hospital economics, the inspection of hospitals, the suggestions of better plans of operating them, and such other matters as may affect the general interests of the membership." This Association was established seven years ago, and began with nine members only. The first two years were mainly social in their results, but in the third year (1901), the membership having increased to forty-four, the Association got to business, and year by year it has developed its activities and increased its field of work to such an extent that in 1905 the number of members had reached 195. There are in the United States some fifteen hundred beneficiary hospitals proper. The scope of the Association has, therefore, great possibilities of development, although it numbers amongst its members at the present time most of the more active and influential medical and lay superintendents of the hospitals in the United States of America.

This Association is in its present form practically the first organisation of the kind which has been established in the history of the world. It may, therefore, be useful to consider the latest of its conferences, held at Boston, Massachusetts, from the point of view of its usefulness and capabilities, having regard to the proceedings thereat and the general results attained. Every meeting of the congress was largely attended, and a majority of the members of the Association, although many of them reside in far distant cities, was present in Boston during the last week in September 1905. To the trained observer one of the most encouraging signs of these meetings of the Conference undoubtedly was the fact that no single one of them was unsuccessful; and that, throughout, the proceedings were characterised by the closest attention, and afforded abundant evidence, not only of the deep personal interest which every member took in giving to the full all the information and experience which each had obtained in the course of his or her official life, but each one in turn undoubtedly derived much information, and gained, too, a better view and grasp of many problems, which could not have been attained with anything like the same facility and completeness in any other way.

Of course there were differences of opinion, and sometimes these differences were sharply and clearly defined, but from first to last the debates were carried on in the most judicial and happy spirit, and all seemed to regard them as a means to be utilised for exploding wrong theories, and confirming the soundest principles, with due regard to the actual facts and circumstances, a result only possible by combining the experience and knowledge of all who took part in the proceedings each day. We have attended many conferences and conventions in regard to hospitals and medical matters during the last thirty years. It is, therefore, but just to Dr. Rowe and the members of the American Association of Hospital Superintendents to state that few, if any, similar gatherings have been more informing, and that none are likely to prove more fruitful in results in the direction of improving and extending the efficiency of the administration of the great medical institutions of the United States, and so indirectly, through the publication of its proceedings, to yield good fruit by raising the average standard of hospital work everywhere.

### Hospital Construction and Architecture.

The morning of the first day, September 26, was devoted to an address of welcome by Dr. David W. Cheever, the eminent surgeon of Boston, and to an address by the President, Dr. George H. M. Rowe, which contained many interesting statistics. The latter we hope to publish in due course. The proceedings closed with a general discussion, in which Sir Henry Burdett, Drs. Hurd, Fisher and Howard, Mr. George P. Ludlam, and others took part. The afternoon of Tuesday was devoted to the consideration of problems relating to hospital construction, including a paper on "Hospital Operating-rooms and their Accessories," by Mr. Edmund M. Wheelwright, architect, and "On the Standardising of Hospital Construction and Equipment," by another Boston architect, Mr. Bertrand E. Taylor. It will be seen from the discussion which followed that excessive outlay upon operating theatres was condemned with practical unanimity, and that the meeting favoured simpler and cheaper methods of providing this unit of hospital construction. It was pointed out that to standardise, or attempt to standardise, hospital construction might tend to stop scientific developments, and in any case any such plan was altogether impracticable, for the reason that both requirements and opinions change in regard to treatment and the units of construction. All that it was possible to hope for was that the experience of the hospital world might continue to find that any man who would devote himself to a careful elaboration of any unit would certainly have the satisfaction, if he succeeded, in knowing that that unit might endure and be reproduced in all the best and most up-to-date modern hospitals. We were glad to notice the unanimous opinion which prevailed in favour of urging upon architects the importance of their devoting their technical knowledge and skill to arrive at some common basis of material and design, which would have the tendency to cheapen, whilst it effectually put a stop to the present tendency to continuously increase, the cost of hospital building. We gathered that many of the speakers had not had an opportunity of studying carefully and comparing the plans of recent hospitals, and that many of them did not realise that the ward unit of a modern hospital in these days must include, in addition to the main ward and its appurtenances, smaller wards, with the addition of rooms set apart for the patients' clothes, for the ward linen, for the dressings and stores, and that they would also comprise a drying-room in connection with the lavatory of sufficient size to secure that the mackintoshes and things of this description were properly treated, and kept in a condition which made them always available for use as and when needed.

### Hospital Administration.

On Wednesday the Conference dealt with the subject of hospital administration, which was introduced by Mr. George P. Ludlam, the chairman of the session, in a thoughtful paper indicating various points well worthy of consideration, on which he left the members to advance opinions, and to suggest solutions. The meeting was remarkable from the fact that a paper was read by Dr. S. S. Goldwater, medical superintendent of the Mt. Sinai Hospital of New York, in which he considered "To what Extent has the Increased Cost in Hospital Construction and Equipment been Justified?" Dr. Goldwater was unfortunately unable from illness to be present, but the masterly way in which he grouped his figures and facts, and the amount of labour represented by the paper, combined with its literary merits and powerful exposition of the difficulties and limitations of the problem presented, made it one of the most valuable and interesting papers of the Congress. The paper entitled "An Aid in the Perfecting of Hospital Management," by Dr. Moses Collins, having been read, a discussion followed which we shall report later.

The afternoon of this day was spent in visiting the hospitals in Boston, and it may be well here to remark that in America the term "hospital" includes lunatic asylums. There were twenty-five of these institutions enumerated on the list supplied to the members, who devoted their time to the particular institution or institutions more immediately connected with the field of work in which they were individually engaged.

### Medical and Hospital Libraries.

In the evening a meeting was held at the Boston Medical Library, and Dr. Henry M. Hurd read a most interesting paper on "John Howard's Observations on Hospitals, 1773-1790," and Mrs. Grace Whiting Myers presented a literary cameo of surpassing excellence and charm, entitled "Some Details About Libraries in Hospitals." The Boston Medical Library was founded by Dr. J. R. Chadwick, to whose energy and devotion it owes its existence. Dr. Chadwick was to have read a paper on medical libraries in hospitals, but he died suddenly on the Sunday morning preceding the Congress. His death, which was totally unexpected, cast a gloom over the proceedings and excited universal regret, which found expression in the speeches that were made in the course of the evening.

### Cold Storage and Engineering Economics.

Thursday morning was devoted to hospital engineering problems, including "Cold Storage and Refrigeration in Hospitals," by Dr. Renwick R. Ross, chairman of the session; "Refuse Destroyers and Disinfectants," by Dr. John H. McCollom; and the paper on "The Utilisation of Hospital Waste," by Dr. F. A. Washburn of the Massachusetts General Hospital, which was fruitful in suggestion, and cannot fail to prove of importance to the managers of every hospital who may be fortunate enough to secure a copy. Mr. Jeremiah C. Long, chief engineer of the Boston City Hospital, presented a useful paper on "Engine-room Economics," and the discussion which followed was practical and interesting to all who have the responsibility of conducting one of the larger hospitals with efficiency and economy.

### The Uniform System of Hospital Accounts.

In the afternoon of the same day Dr. C. Irving Fisher and Mr. James R. Coddington, members of a committee appointed by the previous Congress, made lucid statements advocating uniformity in hospital financial reports and statistics, and submitted a number of specimen accounts, which, after a long and interesting discussion, were referred to the same committee, strengthened by the addition of Dr. J. L. Skinner, with instructions to call in the aid, at their discretion, of an eminent accountant, to circulate the forms of accounts, submitted to the various hospital managers, with the request that they would consider them and return them in due course with such suggestions as to alteration and modification as they might consider desirable or necessary.

The annual dinner was held at the Hotel Vendome on the same evening, under the presidency of Dr. George H. M. Rowe, when Dr. Herbert B. Howard acted as toastmaster, and some interesting speeches were made by Mr. Whitman, professor of Harvard University, Sir Henry Burdett, Mr. George P. Ludlam, and the president.

### The Question Box.

On Friday morning the session was devoted to the "Question Box," under the chairmanship of Dr. John M. Peters. This Question Box is an American institution. The members were requested to present their questions in writing not later than the previous evening. This plan gave the chairman an opportunity to arrange the questions, and it proved so successful and instructive that we make no doubt future Conferences will set apart a whole day to this section, to the great practical advantage and instruction of the members and of all who may be present.

Pensions for Hospital Workers.

Sir Henry Burdett delivered an address, in which he gave a brief outline of the early history of nursing and the initial difficulties that obtained in probations for training up to twenty years ago, and compared them with the present abundance of supplies. He contrasted the position of nursing in England and the United States, gave details of two cases which had led to the establishment of the Royal National Pension Fund for Nurses, and briefly gave an outline of that fund, and of the history of an attempt to establish an American pension fund for nurses in 1891, which made no progress for reasons which he explained. Sir Henry insisted on the duty which devolved upon the trustees and managers of hospitals and asylums to provide that every person in their employ who devoted their lives to the care and attendance of the sick should be provided for on a thrift basis against disablement from illness or accident and the infirmities of age. He then advocated strongly the value to hospitals, and to individual members of the public, of encouraging the duty of personal service in the days of health in the cause of the sick.

The proceedings closed with the election of the president and officers for the ensuing year. Mr. George P. Ludlam of the New York Hospital was unanimously elected President. The Congress unanimously elected Sir Henry Burdett an honorary member of the Association, under Article III., Section IV., of the constitution and by-laws.

Results of the Congress.

There can be no doubt that character has most to do with the real and permanent efficiency of the administration of every institution, large or small. The conclusion of the Conference was marked by evidence of the warm personal friendships which the intercourse of its members had led to, and the cordial recognition of the higher aims and wider atmosphere which the more active minds of those present had given to the majority.

There can be no doubt, too, from the notice which the proceedings received at the hands of the Press that this Boston Congress of the Association of Hospital Superintendents must tend to widen the area of interest in hospitals throughout the United States, and be attended with results which should give the maximum of satisfaction to Dr. George H. M. Rowe, to whose administrative ability, unfailing courtesy, business knowledge, and personal charm, the Association is indebted for the most successful, helpful, inspiriting, and enjoyable Congress which the Association has yet held.

We propose to publish next week a summary of the discussions, bringing out the chief points of practical interest which arose.

